# Metabolic Engineering of Potato Carotenoid Content through Tuber-Specific Overexpression of a Bacterial Mini-Pathway

**DOI:** 10.1371/journal.pone.0000350

**Published:** 2007-04-04

**Authors:** Gianfranco Diretto, Salim Al-Babili, Raffaela Tavazza, Velia Papacchioli, Peter Beyer, Giovanni Giuliano

**Affiliations:** 1 Ente per le Nuove Tecnologie, l'Energia e l'Ambiente (ENEA), Casaccia Research Center, Roma, Italy; 2 Faculty of Biology, Universität Freiburg, Freiburg, Germany; Cairo University, Egypt

## Abstract

**Background:**

Since the creation of “Golden Rice”, biofortification of plant-derived foods is a promising strategy for the alleviation of nutritional deficiencies. Potato is the most important staple food for mankind after the cereals rice, wheat and maize, and is extremely poor in provitamin A carotenoids.

**Methodology:**

We transformed potato with a mini-pathway of bacterial origin, driving the synthesis of beta-carotene (Provitamin A) from geranylgeranyl diphosphate. Three genes, encoding phytoene synthase (CrtB), phytoene desaturase (CrtI) and lycopene beta-cyclase (CrtY) from *Erwinia*, under tuber-specific or constitutive promoter control, were used. 86 independent transgenic lines, containing six different promoter/gene combinations, were produced and analyzed. Extensive regulatory effects on the expression of endogenous genes for carotenoid biosynthesis are observed in transgenic lines. Constitutive expression of the *CrtY* and/or *CrtI* genes interferes with the establishment of transgenosis and with the accumulation of leaf carotenoids. Expression of all three genes, under tuber-specific promoter control, results in tubers with a deep yellow (“golden”) phenotype without any adverse leaf phenotypes. In these tubers, carotenoids increase approx. 20-fold, to 114 mcg/g dry weight and beta-carotene 3600-fold, to 47 mcg/g dry weight.

**Conclusions:**

This is the highest carotenoid and beta-carotene content reported for biofortified potato as well as for any of the four major staple foods (the next best event being “Golden Rice 2”, with 31 mcg/g dry weight beta-carotene). Assuming a beta-carotene to retinol conversion of 6∶1, this is sufficient to provide 50% of the Recommended Daily Allowance of Vitamin A with 250 gms (fresh weight) of “golden” potatoes.

## Introduction

Potato (*Solanum tuberosum*) originated in the highlands of South America, where it has been cultivated for over 2.000 years. Nowadays, potato ranks fourth, among the staple foods of mankind, after wheat, rice and maize. Potato production worldwide stands at 293 million tons, of which 36% in developing countries, and covers more than 18 million hectares (http://www.cipotato.org/market/potatofacts/growprod.htm).

Albeit rich in certain micronutrients, such as vitamin C, cultivated potato is extremely poor in provitamin A. The carotenoid content of tubers in most potato cultivars ranges between 0.5 and 2.5 µg/g FW. The main carotenoids are the xanthophylls lutein and violaxanthin, which are devoid of provitamin A activity. The main provitamin A carotenoid, β-carotene, is present only in trace amounts, from undetectable levels in most cultivars and breeding lines, up to 0.03 µg/g FW [1]. Wild potato species, like *Solanum phureja,* can reach high carotenoid levels in the tuber, but only a minor fraction of these carotenoids is β-carotene [2] [3]. Due to yield and palatability problems, these species cover only a marginal portion of the worldwide potato production. A botanically distant species, sweet potato (*Ipomoea batatas*) can reach high carotenoid and beta-carotene levels in the storage root. Sweet potato ranks seventh among staple crops, with 133 million tons produced annually, 117 of which in a single country, China (http://www.cipotato.org/sweetpotato/). Breeding efforts are under way to break the negative genetic linkage between dry matter and β-carotene content in this crop (http://www.harvestplus.org/sweetpotato3.html).

**Figure 1:**
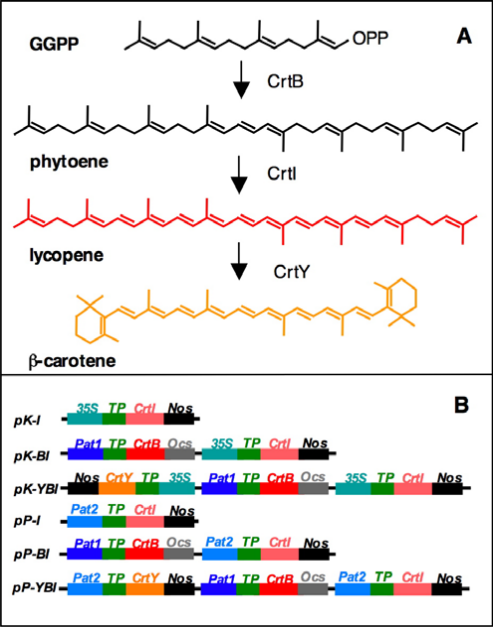
Strategy for the enhancement of the carotenoid content of potato tubers. A: Biosynthetic pathway catalyzed by the CrtB-I-Y genes. B: Schematic representation of the constructs utilized for the transformation experiments. TP: RbcS transit peptide. *Nos* and *Ocs*: Nopaline synthase and Octopine synthase polyadenylation sequences; *35S*: Constitutive CaMV *35S* promoter; *Pat1* and *Pat2*: Tuber-specific patatin promoters. For details, see Materials and Methods.

A large effort has taken place, in the past years, for the metabolic engineering of provitamin A in staple foods of plant origin [4]. Perhaps the best known case is that of “Golden Rice”, produced in a joint effort between one of the laboratories authoring the present paper and the laboratory of Ingo Potrykus [5]. Several other cases are known: in the case of canola and of potato, the seed- and tuber-specific overexpression of the bacterial phytoene synthase, CrtB, causes large increases in both total carotenoids and β-carotene in the target tissues [6] [7]. In tomato, constitutive overexpression of the bacterial phytoene desaturase/isomerase, CrtI, causes β-carotene accumulation and a slight decrease in total carotenoids [8]. In the case of “Golden Rice”, a mini-pathway driving synthesis of β-carotene from geranylgeranyl diphosphate (Figure 1A) has been introduced in rice endosperm (reviewed in [9]). The first step, phytoene synthase (PSY), was of plant origin and was expressed under the control of the endosperm-specific glutelin promoter [5]. The desaturation steps (Figure 1A) were performed by a multifuctional enzyme of bacterial origin, *CrtI*, fused to the RbcS transit peptide and put under the control of the constitutive *35S* promoter [5] [10]. Lycopene cyclase was shown to be dispensible due to sufficient expression of the corresponding endogenous enzyme [5] [11]. Later versions of Golden Rice employed endosperm-specific promoters for all carotenoid transgenes, *PSY* genes from cereals, and have highly increased β-carotene levels (up to 31 µg/g DW) [12]. Codon-optimized versions of the *CrtI* gene have been also tested [13].

In this paper we report the results of a systematic investigation, aimed at verifying which is the optimal combination of promoters and transgenes, able to maximally increase the provitamin A content of potato tubers without affecting vegetative characteristics.

## Results and Discussion

The *Erwinia* genes encoding phytoene synthase (*CrtB*), phytoene desaturase/carotene isomerase (*CrtI*) and lycopene beta-cyclase (*CrtY*) mediate the conversion of geranylgeranyl diphosphate (GGPP) into β-carotene (Figure 1A). The ORFs were fused to the RbcS transit peptide to direct the encoded protein into plastids (Figure 1B). A synthetic *CrtI* gene was used, whose codon usage has been optimized to match that of plants [13]. In all the constructs, the first gene in the pathway (*CrtB*) was put under the control of a tuber-specific *Pat1* promoter, to avoid dwarfism caused by constitutive expression of phytoene synthase [14]. This strategy has been already shown to result in consistent increases of tuber carotenoid and β-carotene content [7]. Genes encoding later steps in the pathway (*CrtI* and/or *CrtY*) were fused to the *35S* promoter in the pK series, or to the tuber-specific *Pat2* promoter in the pP series of constructs. Six constructs, containing different transgene-promoter combinations, were tested (Figure 1B).

Plasmids belonging to the pK series caused lower transformation efficiencies than those belonging to the pP series, indicating that the constitutive expression of CrtY and/or CrtI interfered, to some extent, with the successful establishment of transgenosis. Within the same series, different constructs gave again different efficiencies, in the following order: *CrtI*> *CrtYBI>CrtBI* (Table 1).

**Table 1:** Transformation frequencies

Construct	% regeneration	% PCR-positive regenerants	% Transgenosis
pK-I	61	66	40
pK-BI	24	41	10
pK-YBI	47	37	17
pP-I	73	71	52
pP-BI	29	38	11
pP-YBI	64	57	36

The % of leaf discs giving at least 1 regenerant after 8 weeks on kanamycin is shown in the second column. The % of PCR-positive shoots containing the transgene are shown in the third column. The % transgenosis (fourth column) indicates the % of leaf disks giving at least 1 PCR-positive regenerant.

**Figure 2:**
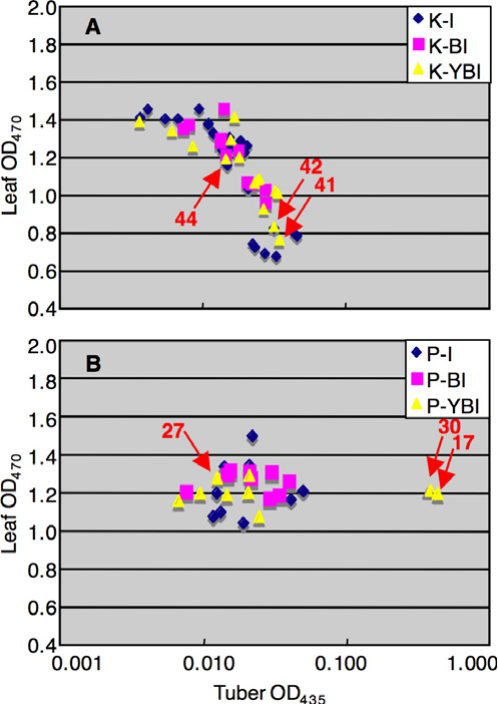
Spectrophotometric quantitation of tuber and leaf carotenoids in transgenic lines. A: Lines transformed with the pK constructs (see Figure 1B). B: Lines transformed with the pP constructs (see Figure 1B). Data are the average of 4 independent tubers from 2 independent plants. Lines submitted to HPLC and Real Time RT-PCR analysis (Tables 2–3 and Figure 4) are indicated by arrows.

The carotenoid content of tubers and leaves of minimum of 9 independent transgenic lines for each construct was analyzed spectrophotometrically, for a total of 86 lines (Figure 2). As can be seen, in plants harboring the pK constructs there is an inverse correlation between tuber and leaf carotenoid content: plants with higher tuber carotenoids have lower leaf carotenoids, and *vice versa* (Figure 2A). Plants harboring the pP constructs, in which the *CrtY* and/or *CrtI* genes are under the control of the *Pat2* promoter, do not show significant changes in leaf carotenoid content (Figure 2B). For most of the constructs, the maximum increase in tuber carotenoids is 3-fold. This is less than what has been obtained with transformation with *Pat:CrtB* alone (4–7 fold) [7].

**Figure 3:**
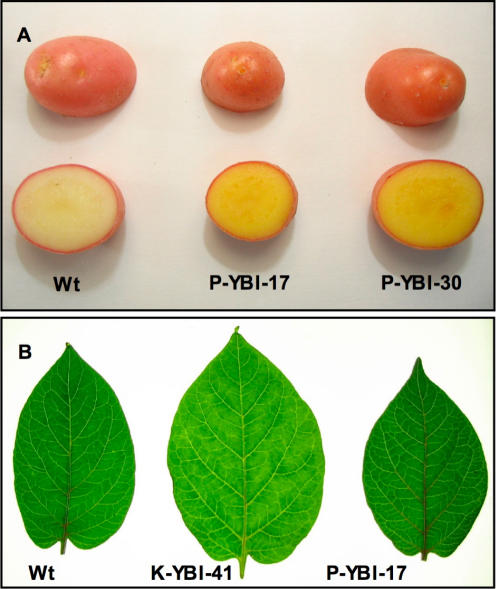
Tuber and leaf phenotypes of transgenic lines. A.Tuber phenotypes. B.Leaf phenotypes, viewed in transmitted light. The difference in size of the middle leaf is not representative.

The pP-YBI construct, in which the whole mini-pathway is expressed under tuber-specific promoter control, stands out from all the other constructs: in two lines (P-YBI 17 and 30), tuber carotenoid content is increased approx. 20-fold, without appreciable effects on leaf carotenoid content (Figure 2B). This increase in tuber carotenoids is much higher than what has been obtained previously with *Pat:CrtB* alone [7]. Tubers of these two lines have a “golden” color (Figure 3A), while their leaves have a normal morphology and pigmentation (Figure 3B). To the opposite, leaves of the *pK* lines with reduced carotenoid content showed signs of chlorosis (Figure 3B).

Our interpretation of these data is that, in the pK constructs, in which the *CrtY* and/or *CrtI* genes are under the control of the 35S promoter, leaf expression of one of the introduced transgenes interferes with leaf carotenogenesis. As a consequence, high expressing lines are counterselected and tuber carotenoid levels show only modest (up to 3-fold) increases. This hypothesis was verified via Real Time RT-PCR on selected transgenic lines (indicated by arrows in Figure 2). The results (Table 2) indicate that the two lines (pK-YBI 41 and 42) showing the highest reduction in leaf carotenoids also show the highest expression of the *CrtI* transcript in leaves, while all the other lines show relatively low expression levels in the same tissue. Changes in carotenoid composition have been observed in leaves expressing the wild-type *CrtI* gene under *35S* promoter control [15] [8]. In the present case, use of a codon-optimized *CrtI* gene probably leads to much higher levels of CrtI protein [13], and thus the potential for interference is higher. In agreement with this model, the pK-YBI 44 line, showing very low levels of transgene expression, has wild-type leaf and tuber carotenoid levels.

**Table 2:** Transgene expression in leaves and tubers

	CrtB		CrtI		CrtY	
	Leaf	Tuber	Leaf	Tuber	Leaf	Tuber
Wild-type	nd	nd	nd	nd	nd	nd
pK-YBI 41	0.0006±0.0001	0.0026±0.0004	0.2094±0.0101	0.1349±0.0195	0.0777±0.0148	0.024±0.0023
pK-YBI 42	0.0003±0.0002	0.0025±0.0004	0.1914±0.0065	0.0865±0.0166	0.0345±0.0109	0.0118±0.0028
pK-YBI 44 (NE)	nd	nd	0.0006±0.0002	0.0013±0.0001	0.0003±0.0001	0.0002±0.0001
pP-YBI 17	0.0004±0.0002	1.1906±0.0882	0.0015±0.0004	12.6922±2.6657	0.0006±0.0001	4.6391±0.0793
pP-YBI 30	0.0005±0.0002	1.1142±0.2328	0.0029±0.0007	3.5258±0.2289	0.0006±0.0002	2.8757±0.1778
pP-YBI 27 (NE)	nd	nd	0.0003±0.0001	0.0018±0.0001	nd	0.0006±0.0003

Values are normalized with respect to the β-tubulin transcript. For each construct, two lines with significant carotenoid changes and one “non expressor” line (NE) are shown.

The carotenoid composition of tubers and leaves from selected lines was analyzed via diode array HPLC. In different harvests, only minor differences in carotenoid content were observed, maintaining the fold increase in carotenoid content over the wild-type and the relative distribution of individual carotenoids. In table 3, we show the mean and standard deviation of carotenoid content from two different harvests.

**Table 3:** HPLC analysis of tuber and leaf pigments (µg/g dry weight)

TUBERS									
Line	Phyt	Alpha	Lutein	Beta	Zea+Anthera	Viola	Neo	Other	Total
Wild-type	0.0	0.0	1.0±0.3	0.013±0.002	1.9±0.5	0.7±0.2	0.6±0.2	1.5±0.6	5.8±1.8
pK-YBI 41	0.0	0.0	2.2±0.6	1.8±0.3	6.6±2.0	0.6±0.2	1.4±0.4	4.2±0.8	16.5±5.2
Fold Variation	–	–	2.2	135.3	3.5	0.8	2.3	2.8	2.8
pK-YBI 42	0.0	0.0	2.0±0.8	1.8±0.3	5.8±2.5	0.6±0.3	2.3±0.8	2.6±1.4	15.2±6.8
Fold Variation	–	–	2.0	142.6	3.1	0.9	3.8	1.7	2.6
pK-YBI 44	0.0	0.0	0.6±0.1	0.0	1.8±0.4	0.4±0.1	0.3±0.1	1.8±0.4	4.9±0.9
Fold Variation	–	–	0.6	–	0.9	0.5	0.5	1.2	0.8
pP-YBI 17	13.8±6.1	6.2±2.4	23.1±5.2	47.4±18.0	11.0±3.7	5.6±1.8	5.7±2.3	1.7±0.6	114.4±41.5
Fold Variation	–	–	23.1	3643.3	5.8	7.9	9.5	1.1	19.7
pP-YBI 30	19.5±1.4	6.1±2.6	30.0±7.3	26.4±4.7	6.2±1.4	22.3±5.7	0.5±0.2	2.1±1.4	112.9±24.9
Fold Variation	–	–	29.9	2031.9	3.3	31.8	0.8	1.4	19.5
pP-YBI 27	0.0	0.0	0.9±0.1	0.0	3.0±0.4	0.4±0.1	0.6±0.2	1.5±0.1	6.4±0.9
Fold Variation	–	–	0.9	–	1.5	0.5	0.9	1.0	1.1
**LEAVES**									
Line	Chl *a*	Chl *b*	Total Chl	Lutein	Beta	Viola	Neo	Other	Total
Wild-type	13846±2038	4343±642	18190±2680	1833±1	1165±1	414±1	617±1	308±1	4337±1
pK-YBI 41	8269±2095	2131±99	10400±2194	1177±418	639±166	238±51	446±106	178±16	2679±757
Fold Variation	0.6	0.5	0.6	0.6	0.5	0.6	0.7	0.6	0.6
pK-YBI 42	8140±1123	2768±221	962±98	962±98	524±66	198±23	368±37	193±89	2245±313
Fold Variation	0.6	0.6	0.6	0.5	0.4	0.5	0.6	0.6	0.5
pK-YBI 44	14169±3241	4619±391	18788±3821	2037±1	1292±1	398±1	631±1	337±1	4695±1
Fold Variation	1.0	1.1	1.0	1.1	1.1	1.0	1.0	1.1	1.1
pP-YBI 17	15208±1593	4800±581	20009±2174	2515±393	1476±230	459±93	691±125	358±76	5500±919
Fold Variation	1.1	1.1	1.1	1.4	1.3	1.1	1.1	1.2	1.3
pP-YBI 30	12674±3377	3865±716	16539±4094	1883±374	869±169	310±45	537±121	245±35	3845±745
Fold Variation	0.9	0.8	0.9	1.0	0.7	0.7	0.9	0.8	0.9
pP-YBI 27	13297±3016	4102±152	17399±4120	1612±106	981±137	348±32	654±73	287±73	3883±422
Fold Variation	1.0	0.9	1.0	0.9	0.8	0.8	1.1	0.9	0.9

Carotenoid composition was measured via diode array HPLC (see Methods) on a minimum of 8 different tubers or leaves from 4 different plants, belonging to 2 different harvests. Fold variation with respect to the wild-type is reported for each carotenoid compound and for each line.

As can be seen, in lines pK-YBI 41 and 42 tuber carotenoids increase, respectively, 2.8 and 2.6-fold. The single carotenoid showing the highest increase is the final product of the introduced mini-pathway, β-carotene, that increases >130-fold. Leaf carotenoid content shows instead a significant decrease, affecting in a comparable fashion all carotenoid species present, as well as Chl *a* and Chl *b.*

“Golden” tubers from lines pP-YBI 17 and 30, in which all three transgenes are under *Patatin* promoter control, show extremely high carotenoid levels, >110 µg/g dry weight. This is at least 3-fold higher than the highest carotenoid content previously reported in potato tubers, including *Pat:CrtB* engineered plants [7], wild accessions like *S. phureja* [2], cultivars carrying the *Orange flesh* gene [1] or transgenic plants carrying the cauliflower *Or* gene [16]. This vast increase in tuber carotenoid content is associated with very high levels of expression (1 to 12-fold tubulin) of the *CrtB-I-Y* transgenes in the tuber, but very low levels of expression of the same transgenes in the leaves (Table 2). Leaf carotenoid content is not affected in “golden” lines (Table 3). β-carotene is the carotenoid species showing the largest increase in “golden” tubers: it increases >3600-fold, to 47 µg/g dry weight, in line pP-YBI 17. This is approx. 5-fold higher than what reported previously [7] and is also the highest β-carotene content reported for the 4 major staple foods of plant origin (wheat, maize, rice, potato), including “Golden Rice 2”, which contains up to 36 µg/g dry weight total carotenoids and 31 µg/g dry weight β-carotene [12]. β-carotene is not the only carotenoid species showing massive increases in these two lines: phytoene, which is undetectable in the Wt line, increases up to 19 µg/g dry weight, indicating that, in spite of the high levels of expression of the codon-optimized *CrtI* transgene, phytoene desaturation is still a rate-limiting step. This finding differs from what has been reported in canola seeds, where overexpression of *CrtI* in conjunction with *CrtB* decreases phytoene to trace levels, with respect to overexpression of *CrtB* alone; another difference between canola and potato is that overexpression of *CrtY* in conjunction with the other two genes does not bring a further increase in total carotenoids [6] [17].

Other carotenoid species show also massive increases in “golden” tubers: α-carotene, which is undetectable in the Wt line, reaches 6 µg/g DW, bringing total provitamin A carotenoids (α- and β-carotene) to 53 µg/g dry weight in line pP-YBI 17. The xanthophylls lutein and violaxanthin, derived, respectively, from α- and β-carotene, increase up to 30-fold (Table 3).

**Figure 4:**
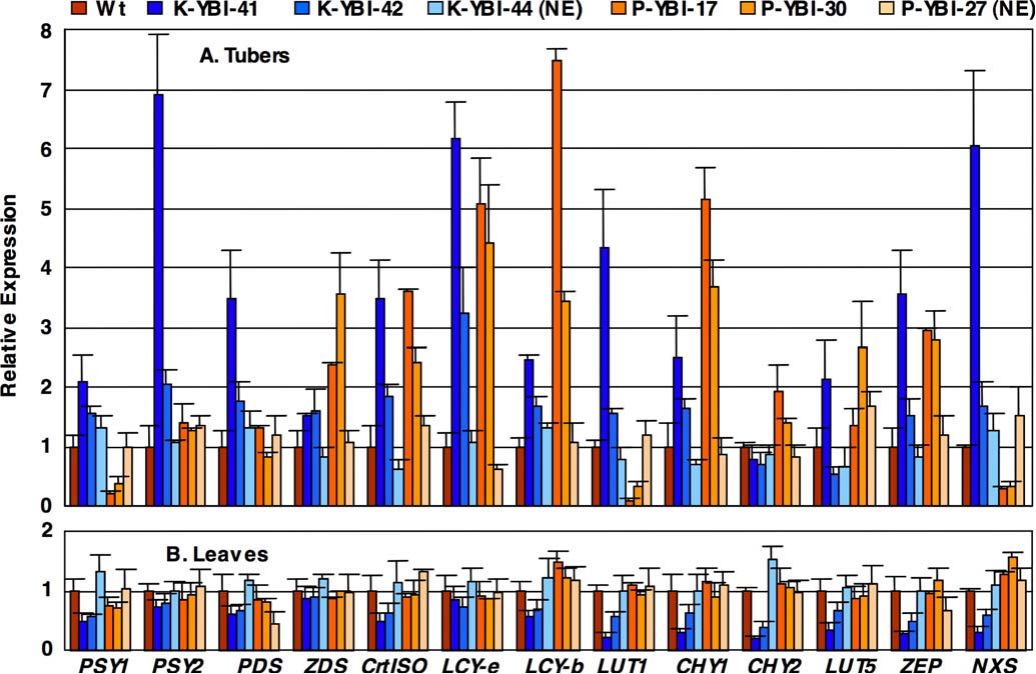
Endogenous carotenoid gene expression. Transcript levels were measured through Real Time RT-PCR and were first normalized for expression of the housekeeping β-tubulin gene, and then for the expression levels in the Wt. A: tubers. B: leaves. For each construct, two lines with significant carotenoid changes and one “non expressor” line (NE) are shown. The histograms show the average and SE (error bars) of determinations from at least 4 different tubers (or leaves) from 2 different plants. For details see Materials and Methods.

Do the reported genetic manipulations have an influence on endogenous carotenoid gene expression? To answer this question, we measured, via Real Time RT-PCR, the expression of genes for the whole carotenoid pathway in tubers and leaves of the Wt and the transgenic lines. The results (Figure 4) can be summarized as follows: in leaves of the lines constitutively expressing *CrtI* and *CrtY* (pK-YBI 41 and 42) a generalized repression is observed, affecting most transcripts, with the exception of *PSY2, ZDS*, and *LCY-e*. Although we are not able to determine whether this is a *cause* or an *effect* of the decrease in leaf carotenoid content, this is a confirmation that constitutive expression of *CrtI* and/or *CrtY* interferes with leaf carotenogenesis; lines expressing *CrtI* and *CrtY* in a tuber-specific fashion (pP-YBI 17 and 30) as well as “non-expressor” lines, show instead only minor perturbations in leaf carotenoid transcripts (Figure 4); in tubers of all the lines analyzed, with the exception of the “non-expressor” lines (pK-YBI 44 and pP-YBI 27), a generalized induction of several transcripts is observed (Figure 4); some notable differences are observed between the pK lines (showing intermediate expression of the transgenes in the tubers) and the pP lines (showing very high transgene expression in this tissue, Table 2).

Thus, by introducing a bacterial mini-pathway for carotenoid biosynthesis, it is possible to increase potato tuber carotenoid content to levels much higher than what has been previously described, either in genetically engineered lines or in natural variants. β−carotene increases to the highest levels described to date in any of the 4 major staple crops. Although different conversion rates of β-carotene into retinol have been reported, this conversion can be as high as 3∶1 [18]. Assuming a more conservative, 6∶1 conversion rate, used in human supplementation studies [19], reaching 50% of the vitamin A RDA of 800 µg retinol equivalents would require 250 gms/day (fresh weight) of the “golden” tubers reported in this work. Although no data exist on the bioavailability of β-carotene from potatoes, it should be noticed that, unlike other antioxidants, carotenoids are not degraded or made less bioavailable after cooking. In fact, cooked sweet potatoes, which are naturally rich in β-carotene, have been shown to improve the vitamin A status of children [20].

Differently from “golden” rice and canola [5] [17], in the case of potato all three genes (*CrtB, CrtI* and *CrtY*) are necessary for attaining maximal levels of tuber carotenoids. Clearly, the “golden” lines seem to be outliers with respect to the majority of lines produced by the pP-YBI construct (Figure 2), indicating that the presence of the construct by itself is not sufficient to give the “golden tuber” phenotype. However, a χ^2^ test shows that this construct produces “golden” lines with a frequency (2/9) significantly different from the double construct pP-BI (0/9, P = 1.4×10^−4^) or the single construct pP-I (0/18, P = 1.3×10^−6^). Finally, it is essential that all three transgenes are put under tuber-specific promoter control, as the expression in leaves of *CrtY* and/or *CrtI* produces a series of alterations in macroscopic phenotypes (Figure 3), biochemical composition (Table 3), and endogenous gene expression (Figure 4).

**Figure 5:**
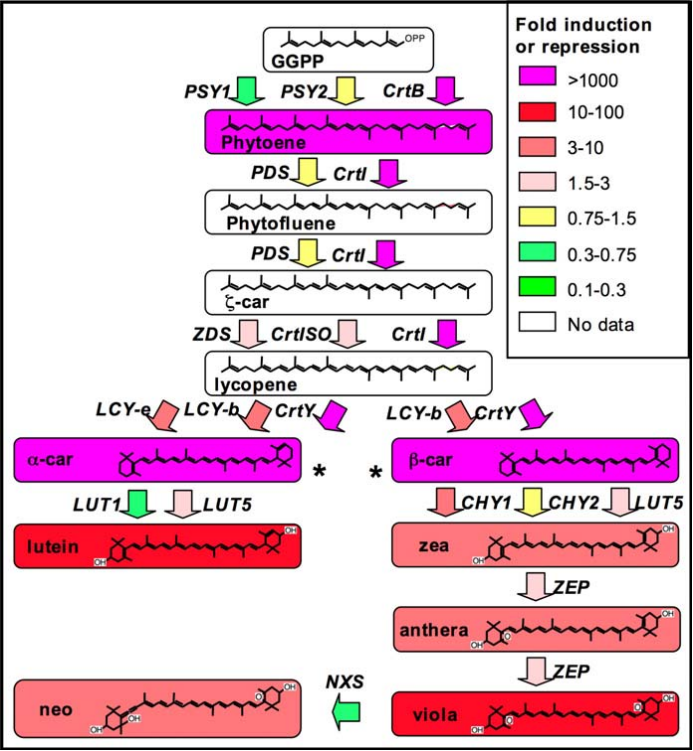
Schematic representation of metabolite and gene expression changes in “golden” tubers. Boxes represent the metabolic intermediates, arrows represent the genes catalyzing the various reactions. Fold induction or repression with respect to the wild-type - averaged over lines P-YBI 17 and 30 - is represented by different color hues (see legend). Asterisks mark Provitamin A carotenoids (α- and β-carotene).

In “golden” tubers, provitamin A carotenoids (α- and β-carotene) increase dramatically (Figure 5) as do a series of downstream compounds (lutein, violaxanthin, neoxanthin). The increase in these compounds is paralleled by the increase in some of the endogenous transcripts involved in their biosynthesis: *CrtISO, LCY-e, CHY1, ZEP* (Figure 5). Some of the observed perturbations are likely to antagonize the accumulation of provitamin A carotenoids: for instance, the induction of *CHY1, LUT5* and *ZEP* favors the partial conversion of α- and β-carotene into α- and β-xanthophylls [21] [22] [23] [24] [25] [26]. In fact, the levels of these xanthophylls increase, although not as dramatically as α- and β-carotene (Figure 5). These xanthophylls may also have a beneficial nutritional effect, since high dietary intakes of xanthophylls, particularly of lutein, have been associated with a lowered risk for certain cancers and eye degenerative syndromes [27].

What about the stability of the “golden tuber” trait? Since potato is propagated vegetatively, rather than sexually, we checked the stability through subsequent generations of micropropagated plants. Each transformed line, derived from a single transformation event, has been multiplied in vitro for over two years, and several different harvests have been tested spectrophotometrically and via HPLC, finding only minor variations in carotenoid content (Table 3).

We believe that the carotenoid and provitamin A content of potato can be further improved, with respect to the results shown here: first, we and others have shown that gene silencing can be exploited to re-direct the biosynthetic flux in the pathway, enhancing the accumulation of specific carotenoids such as β-carotene or zeaxanthin [28] [29] [26]; second, the accumulation of phytoene observed in the “golden” tubers indicates that, in spite of the expression of a codon-optimized *CrtI* gene, phytoene desaturation is still a rate-limiting step; third, Lu et al. [16] have shown that the cauliflower *Or* gene can be used to increase total carotenoid and β-carotene levels in tubers; since this gene does not appear to increase the capacity for biosynthesis [30], which is the main target of the manipulation reported in this work, it is possible that it will have an additive affect with respect to the constructs reported here. Therefore, a combination of different approaches is very promising for the further improvement of potato carotenoid content, either for nutritional purposes, or for the production of high value-added compounds.

## Materials and Methods

Unless indicated differently, molecular biology methods are as described [31]. The *Pat1* promoter has been described previously [32] [29], while the *Pat2* promoter is a 648-bp fragment from the P24 promoter [33] flanked by Sal I (upstream) and Bam HI (downstream) restriction sites. *CrtB* and *CrtY* genes are from *Erwinia herbicola* [34]. The synthetic *CrtI* gene [13] and the RbcS transit peptide [15] have been described. The transformation vectors are shown in Figure 1B and they are derivatives of pCAMBIA 1390 [35]. They were constructed as follows:

pK-I: The synthetic *TP-CrtI* gene was excised from pFun1 [13] using BamHI and ligated into a pCAMBIA1390 derivative containing the CaMV *35S* promoter and a kanamycin resistance gene to yield pK-I.

pK-BI: To generate a *TP-CrtB* fusion, the *CrtB* gene was amplified from the *Erwinia herbicola* gene cluster using the following primers: CrtB-Rev: 5′- GATTGAGGCATGCCAATGAGCCA-3′ and CrtB-For: 5′-ACCTACAGGGGTACCTGCGTGA-3′. The obtained fragment was then digested with KpnI, filled-in with the T4-DNA-polymerase, treated with SphI and finally ligated into pTBlue, a pBluescript derivative encoding the RbcS transit peptide [15] and harboring an additional polylinker from pUC18, to yield pTCrtB. The *TP-CrtB* gene was then isolated through KpnI-digestion and ligated into pAsBB33, a pBluescript derivative harboring the patatin 1 promoter, the *ocs* terminator and a pUC18 polylinker flanked by two AscI-sites. The *TP-CrtB* expression cassette was then excised from the obtained plasmid, pTCrtB33, using AscI and ligated into the corresponding site in pK-I to yield pK-BI.

pK-YBI: *CrtY* was amplified from the *Erwinia herbicola* gene cluster using the following primers: CrtY-Rev: 5′-GAGAGCGTAGCATGCGGGATCTGA-3′and CrtY-For: 5′-AGCTCGAGGATCCACCAAAGCCTG-3′. The obtained fragment was then digested with SphI/XhoI and ligated into the corresponding sites of pTBlue to create a *TP-CrtY* fusion gene. The *TP-CrtY* fragment was then isolated from the generated plasmid, pTCrtY, using BamHI and XbaI and ligated into the corresponding sites of pUCET4 [15] to yield pUCrtY. The generated *TP-CrtY* expression cassette was then isolated from pUCrtY as a blunt-end fragment through EcoRI/HindIII-digestion and subsequent T4-DNA-polymerase treatment. The fragment was then ligated into EcoRI-digested and blunt-ended pTCrtB33 to yield pTCrtBY33. The fragment encoding the *TP-CrtB* and *TP-CrtY* expression cassettes was then isolated from pTCrtBY33 using AscI and ligated into the corresponding site of pK-I to yield pK-YBI

pP-I: The synthetic *TP-CrtI* gene was excised from pK-I as a BamHI-fragment and ligated into the corresponding site of pPAT1390, a pCAMBIA1390 derivative containing the patatin 2 promoter and a kanamycin resistance gene, to yield pP-I.

pP-BI: The *TP-CrtB* expression cassette was excised from pTCrtB33 using AscI and ligated into the corresponding site of pP-I to yield pP-BI.

pP-YBI: The patatin 2 promoter was ligated as HindIII/SmaI fragment into XbaI-digested, T4-DNA-polymerase- and HindIII-treated pUCrtY replacing the 35S CaMV promoter to yield pPAT-TCrtY. To isolate the *TP-CrtY* expression cassette, pPAT-TCrtY was then digested with HindIII/EcoRI and filled-in with the T4-DNA-polymerase. The obtained fragment was then ligated into EcoRI-digested and T4-DNA-polymerase-treated pTCrtB33 to yield pPAT-CrtBY. The fragment encoding the *TP-CrtB* and *TP-CrtY* expression cassettes was then isolated from pPAT-CrtBY using AscI and ligated into the corresponding site of pP-I to yield pP-YBI.

Potato (cv Desirée) was transformed and transformants were selected on kanamycin and tested via PCR as described previously [36] [29]. In order to select for independent transformation events, only one regenerant/leaf disk was isolated. PCR primers were: *CrtB* For: CTG ACC CAC GGT ATT ACG; *CrtB* Rev: CGT CTT CGC CCG AAT AAC; *CrtI* For: GCG ACC AGT AGC ATC TAC; *CrtI* Rev: GTT AGA TGC CAC GGC TTG; *CrtY* For: CAT TCC ATG AAG ACG ATC TG; *CrtY* Rev: GCG AAT AGC CAG TGG TAG. Each line was micropropagated [36] and two plantlets were adapted and brought to maturity in the greenhouse [29]. All carotenoid and RT-PCR measurements were conducted on at least 4 different “deep” tubers derived from the 2 plants, to account for plant-to-plant variation and to minimize possible alterations in carotenoid composition/gene expression resulting from light accidentally illuminating the superficial tubers.

RNA isolation, Real Time RT-PCR conditions and primers were described previously [29]. Additional primers used in this work were: *Nxs* For: CTTGGAGGAGACTTCTTTGGTGA; *Nxs* Rev: CGGAAGTGGTCCTCCCATAG; *Lut5* For: GTCTCAAGCAAGCAACTTCGTG; *Lut5* Rev: GATAAAAGGTCCATGTGAGCACTG;

For spectrophotometric quantitation of carotenoids, tuber samples (∼1.5–2.0 g FW) were peeled and homogenized with 2 ml of water with an Ultraturrax homogenizer at full speed until they were completely disrupted; 2 ml of acetone were added and the homogenization was continued for 30”. 2 ml of light Petroleum Ether (PE) were added to each sample and, after vortexing for 2′, the samples were centrifuged at 8000× g for 5′ in a swinging bucket rotor. The 400–500 nm spectrum of the upper phase (PE+carotenoids) was determined against a PE blank. Leaf pigment quantitation was performed as described previously [37].

For HPLC analysis, frozen tubers were peeled, lyophilized, ground to powder and pigments were extracted three times with 2 ml of acetone. In the first extraction, 200 µg tocopherol acetate per sample was added as an internal standard. Combined acetone extracts were dried, lipophilic compounds were resuspended in 2 ml of petroleum ether:diethyl ether (2∶1, v/v) and 1 ml of distilled water. After centrifugation for 5 min at 3000× g the organic phase was recovered and the aqueous phase was extracted for a second time as described above. The combined organic phases were dried and dissolved in 30 µl chloroform. 10 µl were subjected to HPLC analysis with a C30 reversed-phase column (YMC Europe GmbH, Schermbeck, Germany) and a gradient system as described [10]. Carotenoids were identified by their absorption spectra, monitored using a photodiode array detector (PDA 2996; Waters, Eschborn, Germany). Peak areas of individual carotenoids were integrated electronically at the respective λ_max_. Total carotenoid levels in different lines were normalized using spectrophotometry.

## References

[1] Nesterenko S, Sink KC (2003). Carotenoid profiles of potato breeding lines and selected cultivars.. HortScience.

[2] Morris WL, Ducreux L, Griffiths DW, Stewart D, Davies HV (2004). Carotenogenesis during tuber development and storage in potato.. J Exp Bot.

[3] Griffiths DW, Dale MF, Morris WL, Ramsay G (2007). Effects of season and postharvest storage on the carotenoid content of Solanum phureja potato tubers.. J Agric Food Chem.

[4] Giuliano G, Aquilani R, Dharmapuri S (2000). Metabolic engineering of plant carotenoids.. Trends Plant Sci.

[5] Ye X, Al-Babili S, Kloti A, Zhang J, Lucca P (2000). Engineering the provitamin A (beta-carotene) biosynthetic pathway into (carotenoid-free) rice endosperm.. Science.

[6] Shewmaker CK, Sheehy JA, Daley M, Colburn S, Ke DY (1999). Seed-specific overexpression of phytoene synthase: increase in carotenoids and other metabolic effects.. Plant J.

[7] Ducreux LJ, Morris WL, Hedley PE, Shepherd T, Davies HV (2005). Metabolic engineering of high carotenoid potato tubers containing enhanced levels of beta-carotene and lutein.. J Exp Bot.

[8] Romer S, Fraser PD, Kiano JW, Shipton CA, Misawa N (2000). Elevation of the provitamin A content of transgenic tomato plants.. Nat Biotechnol.

[9] Al-Babili S, Beyer P (2005). Golden Rice–five years on the road–five years to go?. Trends Plant Sci.

[10] Hoa TT, Al-Babili S, Schaub P, Potrykus I, Beyer P (2003). Golden Indica and Japonica rice lines amenable to deregulation.. Plant Physiol.

[11] Schaub P, Al-Babili S, Drake R, Beyer P (2005). Why is golden rice golden (yellow) instead of red?. Plant Physiol.

[12] Paine JA, Shipton CA, Chaggar S, Howells RM, Kennedy MJ (2005). Improving the nutritional value of Golden Rice through increased pro-vitamin A content.. Nat Biotechnol.

[13] Al-Babili S, Hoa TT, Schaub P (2006). Exploring the potential of the bacterial carotene desaturase CrtI to increase the beta-carotene content in Golden Rice.. J Exp Bot.

[14] Fray RG, Wallace A, Fraser PD, Valero D, Hedden P (1995). Constitutive expression of a fruit phytoene synthase gene in transgenic tomatoes causes dwarfism by redirecting metabolites from the gibberellin pathway.. Plant J.

[15] Misawa N, Yamano S, Linden H, de Felipe MR, Lucas M (1993). Functional expression of the Erwinia uredovora carotenoid biosynthesis gene crtl in transgenic plants showing an increase of beta-carotene biosynthesis activity and resistance to the bleaching herbicide norflurazon.. Plant J.

[16] Lu S, Van Eck J, Zhou X, Lopez AB, O'Halloran DM (2006). The Cauliflower Or Gene Encodes a DnaJ Cysteine-Rich Domain-Containing Protein That Mediates High-Levels of {beta}-Carotene Accumulation.. Plant Cell.

[17] Ravanello MP, Ke D, Alvarez J, Huang B, Shewmaker CK (2003). Coordinate expression of multiple bacterial carotenoid genes in canola leading to altered carotenoid production.. Metab Eng.

[18] Howe JA, Tanumihardjo SA (2006). Carotenoid-biofortified maize maintains adequate vitamin a status in Mongolian gerbils.. J Nutr.

[19] West KP, Katz J, Khatry SK, LeClerq SC, Pradhan EK (1999). Double blind, cluster randomised trial of low dose supplementation with vitamin A or beta carotene on mortality related to pregnancy in Nepal. The NNIPS-2 Study Group.. Bmj.

[20] van Jaarsveld PJ, Faber M, Tanumihardjo SA, Nestel P, Lombard CJ (2005). Beta-carotene-rich orange-fleshed sweet potato improves the vitamin A status of primary school children assessed with the modified-relative-dose-response test.. Am J Clin Nutr.

[21] Duckham SC, Linforth RST, Taylor IB (1991). Abscisic-acid-deficient mutants at the *aba* gene locus of *Arabidopsis thaliana* are impaired in the epoxidation of zeaxanthin.. Plant Cell Environ.

[22] Tian L, Magallanes-Lundback M, Musetti V, DellaPenna D (2003). Functional analysis of beta- and epsilon-ring carotenoid hydroxylases in Arabidopsis.. Plant Cell.

[23] Tian L, Musetti V, Kim J, Magallanes-Lundback M, DellaPenna D (2004). The Arabidopsis LUT1 locus encodes a member of the cytochrome p450 family that is required for carotenoid epsilon-ring hydroxylation activity.. Proc Natl Acad Sci U S A.

[24] Kim J, DellaPenna D (2006). Defining the primary route for lutein synthesis in plants: the role of Arabidopsis carotenoid beta-ring hydroxylase CYP97A3.. Proc Natl Acad Sci U S A.

[25] Fiore A, Dall'osto L, Fraser PD, Bassi R, Giuliano G (2006). Elucidation of the beta-carotene hydroxylation pathway in Arabidopsis thaliana.. FEBS Lett.

[26] Diretto G, Welsch R, Tavazza R, Mourgues F, Pizzichini D (2007). Silencing of beta-carotene hydroxylase increases total carotenoid and beta-carotene levels in potato tubers.. BMC Plant Biol.

[27] Krinsky NI (2002). Possible biological mechanisms for a protective role of xanthophylls.. J Nutr.

[28] Romer S, Lubeck J, Kauder F, Steiger S, Adomat C (2002). Genetic engineering of a zeaxanthin-rich potato by antisense inactivation and co-suppression of carotenoid epoxidation.. Metab Eng.

[29] Diretto G, Tavazza R, Welsch R, Pizzichini D, Mourgues F (2006). Metabolic engineering of potato tuber carotenoids through tuber-specific silencing of lycopene epsilon cyclase.. BMC Plant Biol.

[30] Li L, Lu S, Cosman KM, Earle ED, Garvin DF (2006). beta-Carotene accumulation induced by the cauliflower Or gene is not due to an increased capacity of biosynthesis.. Phytochemistry.

[31] Sambrook J, Fritsch EF, Maniatis T (1989). Molecular Cloning. A Laboratory Manual (Second Edition).

[32] Rocha-Sosa M, Sonnewald U, Frommer W, Stratmann M, Schell J (1989). Both developmental and metabolic signals activate the promoter of a class I patatin gene.. Embo J.

[33] Liu XY, Rocha-Sosa M, Hummel S, Willmitzer L, Frommer WB (1991). A detailed study of the regulation and evolution of the two classes of patatin genes in Solanum tuberosum L.. Plant Mol Biol.

[34] To KY, Lai EM, Lee LY, Lin TP, Hung CH (1994). Analysis of the gene cluster encoding carotenoid biosynthesis in Erwinia herbicola Eho13.. Microbiology.

[35] Hajdukiewicz P, Svab Z, Maliga P (1994). The small, versatile pPZP family of Agrobacterium binary vectors for plant transformation.. Plant Mol Biol.

[36] Tavazza R, Tavazza M, Ordas RJ, Ancora G, Benvenuto E (1988). Genetic transformation of potato (*Solanum tuberosum*); an efficient method to obtain transgenic plants.. Plant Science.

[37] Porra RJ, Thompson WA, Kriedermann PE (1989). Determination of accurate extinction coefficients and simultaneous equation for assaying chlorophyll a and b extracted with four different solvents: verification of the concentration of chlorophyll standards by atomic absorption spectroscopy.. Biochim Biophys Acta.

